# Geography and Location Are the Primary Drivers of Office Microbiome Composition

**DOI:** 10.1128/mSystems.00022-16

**Published:** 2016-04-19

**Authors:** John Chase, Jennifer Fouquier, Mahnaz Zare, Derek L. Sonderegger, Rob Knight, Scott T. Kelley, Jeffrey Siegel, J. Gregory Caporaso

**Affiliations:** a Department of Biological Sciences, Northern Arizona University, Flagstaff, Arizona, USA; b Center for Microbial Genetics and Genomics, Northern Arizona University, Flagstaff, Arizona, USA; c Department of Biology, San Diego State University, San Diego, California, USA; d Department of Civil Engineering, University of Toronto, Toronto, Ontario, Canada; e Department of Mathematics and Statistics, Northern Arizona University, Flagstaff, Arizona, USA; f Department of Computer of Science, University of California San Diego, San Diego, California, USA; g Department of Pediatrics, University of California San Diego, San Diego, California, USA; h Dalla Lana School of Public Health, University of Toronto, Toronto, Ontario, Canada; Argonne National Laboratory

**Keywords:** bacteria, built environment, fungi, microbiome

## Abstract

In the United States, humans spend the majority of their time indoors, where they are exposed to the microbiome of the built environment (BE) they inhabit. Despite the ubiquity of microbes in BEs and their potential impacts on health and building materials, basic questions about the microbiology of these environments remain unanswered. We present a study on the impacts of geography, material type, human interaction, location in a room, seasonal variation, and indoor and microenvironmental parameters on bacterial communities in offices. Our data elucidate several important features of microbial communities in BEs. First, under normal office environmental conditions, bacterial communities do not differ on the basis of surface material (e.g., ceiling tile or carpet) but do differ on the basis of the location in a room (e.g., ceiling or floor), two features that are often conflated but that we are able to separate here. We suspect that previous work showing differences in bacterial composition with surface material was likely detecting differences based on different usage patterns. Next, we find that offices have city-specific bacterial communities, such that we can accurately predict which city an office microbiome sample is derived from, but office-specific bacterial communities are less apparent. This differs from previous work, which has suggested office-specific compositions of bacterial communities. We again suspect that the difference from prior work arises from different usage patterns. As has been previously shown, we observe that human skin contributes heavily to the composition of BE surfaces.

**IMPORTANCE** Our study highlights several points that should impact the design of future studies of the microbiology of BEs. First, projects tracking changes in BE bacterial communities should focus sampling efforts on surveying different locations in offices and in different cities but not necessarily different materials or different offices in the same city. Next, disturbance due to repeated sampling, though detectable, is small compared to that due to other variables, opening up a range of longitudinal study designs in the BE. Next, studies requiring more samples than can be sequenced on a single sequencing run (which is increasingly common) must control for run effects by including some of the same samples in all of the sequencing runs as technical replicates. Finally, detailed tracking of indoor and material environment covariates is likely not essential for BE microbiome studies, as the normal range of indoor environmental conditions is likely not large enough to impact bacterial communities.

## Abstract (precis)

Our study highlights several points that should impact the design of future studies of the microbiology of BEs. First, projects tracking changes in BE bacterial communities should focus sampling efforts on surveying different locations in offices and in different cities but not necessarily different materials or different offices in the same city. Next, disturbance due to repeated sampling, though detectable, is small compared to that due to other variables, opening up a range of longitudinal study designs in the BE. Next, studies requiring more samples than can be sequenced on a single sequencing run (which is increasingly common) must control for run effects by including some of the same samples in all of the sequencing runs as technical replicates. Finally, detailed tracking of indoor and material environment covariates is likely not essential for BE microbiome studies, as the normal range of indoor environmental conditions is likely not large enough to impact bacterial communities.

## INTRODUCTION

In the United States, humans spend over 90% of their time in built environments (BEs) (1, 2) such as homes, offices, hospitals, and cars. We know that microbes in the BE affect human health (3–5) and the rate of degradation of building materials (6, 7). However, until recently, very little was known about the microorganisms that cohabit with us in these environments. Over the past decade, molecular microbial diversity studies have shed new light on the spatial and temporal variations of microbial communities in BEs (8–11). Recent work has revealed how microbial communities, or microbiomes, differ with different building systems (e.g., ventilation mechanisms) (5), how new buildings are colonized by microorganisms (11, 12), and how the human microbiome both impacts and is impacted by the microbiome of a home (13). Differences in microbiomes have been reported across different BE spaces (8, 10, 13), suggesting that our offices and homes have individualized microbiomes, and it has been suggested that microbiome composition differs on the basis of the surface material where the community is found (13–15).

Prior work has not directly tested whether variation in BE microbiomes is due mainly to the geographic location, the material that is being sampled, the location in the room that is being sampled, the specific inhabitants, or the environmental conditions that exist within a given indoor environment, all of which have been noted as potential sources of variation but are difficult to separate. This study aimed to expand our basic understanding of the microbiology of BEs by separating factors that are often conflated in BE studies, such as the surface material type (e.g., ceiling tile) and the location in an office (e.g., the ceiling), to understand how factors independently contribute to the composition of BE microbiomes. Similarly, we aimed to understand which, if any, indoor or material microenvironment parameters, such as temperature or humidity, are associated with differences in office microbiomes. Finally, we wished to understand how the human microbiome of an office’s inhabitants relates to the personalized office microbiome effect that has previously been suggested (14) and specifically whether this effect extends to surfaces that office inhabitants are not in direct contact with.

Determining the sources of variation in BE microbiomes requires that multiple offices be evaluated and that multiple locations, material types, and human inhabitants of each office be sampled. Further, if office microbiomes differ geographically, we must survey across a wide enough geographic range that climatic differences are apparent in the indoor environments. We therefore monitored nine offices, three each in Flagstaff, AZ; San Diego, CA; and Toronto, ON, and collected microbiome samples over the course of a year, along with dense indoor environment data such as temperature, occupancy, and humidity. If office microbiomes differ because of material and/or location in the office, we need to be able to separate these variables to determine their relative contributions. Accordingly, we installed carpet, ceiling tile, and drywall swatches on the floor, wall, and ceiling. Microbiome samples were collected in four 6-week intensive sampling periods in the summer, fall, winter, and spring, and some material swatches were sampled more frequently than others. Finally, we collected human microbiome samples from the individuals who performed the sampling and from 11 inhabitants of one of the offices.

Our design allowed us to evaluate and distinguish the impacts of the building material, location in the office, sampling frequency, city, office, time, and indoor environment covariates on the bacterial communities that established themselves on each of the sample swatches. Our findings provide information on the factors associated with office microbiome composition under normal circumstances and provide essential information for informed experimental design in future studies of the microbiology of BEs.

## RESULTS

### Experimental design.

**FIG 1:**
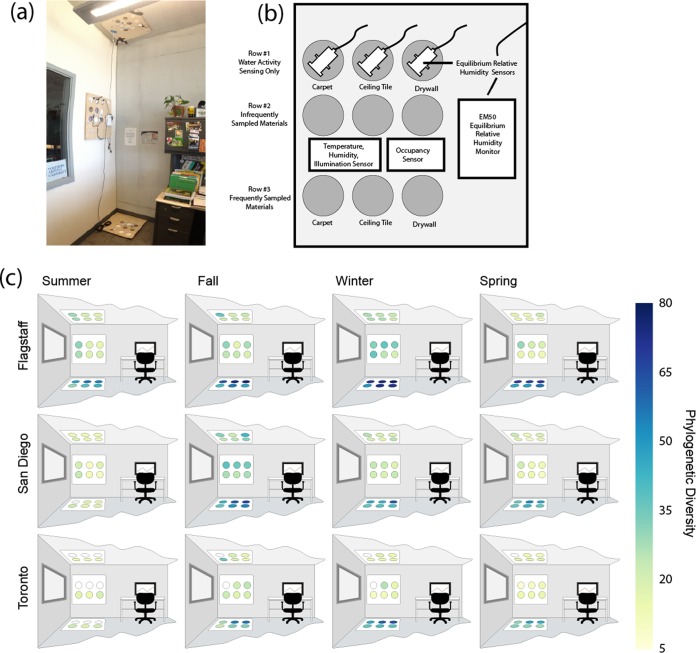
Experimental design. (a) Configuration of the sampling site in Flagstaff office 1. This configuration was similar to those set up in all of the offices. Signs on the wall adjacent to the wall sampling plate describe the project and request that the materials not be touched. (b) Diagram of a single sampling plate illustrating nine sampling swatches (circles) of three different materials, one row for tracking the equilibrium relative humidity of the materials (row 1), one row for infrequent sampling (row 2), and one row for frequent sampling (row 3). (c) Samples were collected from rows 2 and 3 of all of the sampling plates in three offices in each of our three cities in four intensive sampling periods over the course of 1 year. The coloring of the sampling swatches illustrates the change in bacterial PD over the year.

To develop our understanding of how microbes establish themselves in BEs over time and a range of building parameters, we sampled nine offices in three cities over a 1-year period. The selected cities, San Diego, CA; Flagstaff, AZ; and Toronto, ON, are climatically different from one another. Within each city, we chose three offices that were as similar to each other as possible (details of our selection and exclusion criteria are provided in Materials and Methods; for details of the parameters of our offices, see Table S7 in the supplemental material). In each office, we installed three sampling plates, with one plate each on the floor, ceiling, and wall, as illustrated in Fig. 1a (also see Fig. S1 in the supplemental material). Each plate contained two or three swatches each of painted drywall, ceiling tile, and carpet, allowing us to dissociate the location in the room from the material, as well as sensors that allowed us to monitor parameters of the environment including equilibrium relative humidity on the surfaces of the swatches, available light, occupancy, temperature, and relative humidity (Fig. 1b). Samples were collected in four 6-week sampling periods, one per season. Bacterial and fungal genetic markers were sequenced from these samples by using 16S rRNA gene sequencing to profile bacterial communities and internal transcribed spacer 1 (ITS1, the noncoding region between the 18S and 5.8S rRNA genes) sequencing to profile fungal communities. Our analysis focused on bacterial data, as we obtained less usable data for fungi, which we suspect may be a result of the low biomass of our samples. For the results of our fungal sequencing, see Text S4 in the supplemental material.

10.1128/mSystems.00022-16.1 Figure S1  Experimental design. This is an analog of Fig. 1 illustrating fungal community results. Download Figure S1, TIF file, 5.6 MB. Copyright © 2016 Chase et al. 2016 Chase et al. This content is distributed under the terms of the Creative Commons Attribution 4.0 International license.

10.1128/mSystems.00022-16.4 Text S4  Results of fungal community profiling, corresponding methods, and greater detail of experiments performed to explore associations between office microbiome composition and indoor and material environment covariates. Download Text S4, DOCX file, 0.02 MB. Copyright © 2016 Chase et al. 2016 Chase et al. This content is distributed under the terms of the Creative Commons Attribution 4.0 International license.

10.1128/mSystems.00022-16.7 Table S7  Descriptions of the offices included in this study. Download Table S7, XLSX file, 0.05 MB. Copyright © 2016 Chase et al. 2016 Chase et al. This content is distributed under the terms of the Creative Commons Attribution 4.0 International license.

### Location in office, but not building material, drives community composition.

This study was designed so that each material (drywall, carpet, or ceiling tile) was installed at each location (ceiling, floor, or wall) in every office in this study. This design allowed us to differentiate the roles that the location in an office and the surface material play in determining the richness (how many types of organisms are present) and composition (which taxa are present or absent and their relative abundances) of bacterial communities on those surfaces.

**FIG 2:**
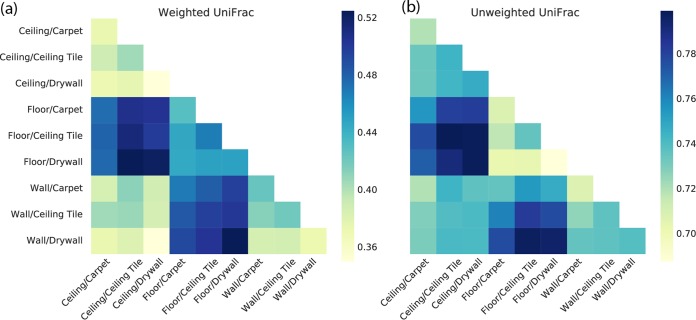
Bacterial community dissimilarity, as measured by weighted UniFrac, a quantitative measurement (a), and unweighted UniFrac, a qualitative measurement (b), of the Flagstaff samples (similar patterns were observed across all of the cities). Darker colors indicate that groups of samples are more dissimilar, and lighter colors indicate that groups of samples are more similar. The labels indicate the locations in the office, followed by the material type. For example, ceiling/carpet indicates the samples of carpet that are installed on the ceiling sampling plate, and investigation of the first column of panel a shows that the carpet samples on the ceiling are more similar to the carpet samples on the wall than they are to the carpet samples on the floor.

**FIG 3:**
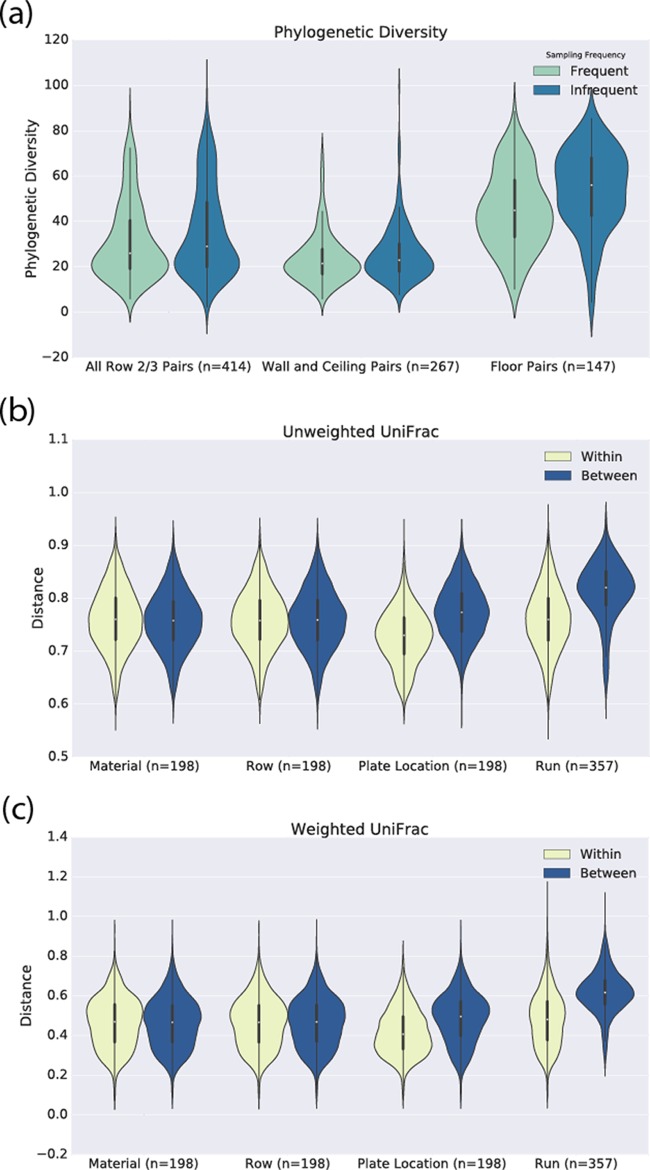
Disturbance due to repeat sampling, though detectable, is small compared to that due to other variables. (a) The PD of frequently sampled swatches (row 2, see Fig. 1b) was consistently lower than the PD of the frequently sampled swatches, suggesting that sampling decreases the PD of the sites. (b) Weighted UniFrac shows that samples with the same sampling frequency are more similar to each other than samples with different sampling frequencies are, suggesting an effect of repeat sampling. However, comparison of this difference to effects of biological interest show that the sampling frequency has a larger effect on the bacterial communities than the material does (which, as shown in Fig. 2, is effectively null) but a smaller effect than the location of the plate in the office and the sequencing run, which are shown in Fig. 2 and 5 to impact our observed bacterial communities. (c) Similar results were obtained with unweighted UniFrac.

With unweighted and weighted UniFrac (16), qualitative and quantitative measurements of microbial community dissimilarity, respectively, the material was not observed to be a significant driver of bacterial community composition (Fig. 2 and 3). Similarly, with Faith’s phylogenetic diversity (PD) (17), a measurement of the phylogenetic richness of a community, the material did not appear to be a driver of richness (Fig. 1c). The location within an office where a sample was collected, however, was associated with both community richness (Fig. 1c and 3) and community composition (Fig. 2; see Fig. S2 in the supplemental material). Floor samples were significantly richer than either ceiling or wall samples in all of the cities across all of the sampling periods, except for the first sampling period (two-sample Monte Carlo *t* test with 999 permutations, *P* < 0.05 for all comparisons). We suspect that the lack of difference in the first sampling period is due to the recent sterilization of the materials (see Materials and Methods). There were no statistically significant differences in community richness between wall and ceiling samples. This observation is consistent with the higher deposition rates of larger particles and dust to upward-facing horizontal surfaces like floors.

10.1128/mSystems.00022-16.2 Figure S2  Fungal community dissimilarity. This is an analog of Fig. 2 illustrating fungal community results. Download Figure S2, TIF file, 5 MB. Copyright © 2016 Chase et al. 2016 Chase et al. This content is distributed under the terms of the Creative Commons Attribution 4.0 International license.

We focused our analysis of surface material and location effects on community composition on the Flagstaff samples taken during the fall and winter sampling periods, which were all sequenced in the same sequencing run to avoid the sequencing run being a variable in this analysis. These samples showed significant differences in community composition by the location that was sampled in the office (permutational multivariate analysis of variance [PERMANOVA] with weighted UniFrac pseudo-*F* = 7.80, *P* < 0.0001; unweighted UniFrac pseudo-*F* = 3.54, *P* < 0.0001), but not by the surface material (PERMANOVA with weighted UniFrac pseudo-*F* = 1.17, *P* = 0.22, unweighted UniFrac pseudo-*F* = 1.01, *P* = 0.89). As with community richness, the floor samples were more different from the wall and ceiling samples, which were virtually indistinguishable from one another. For the specific taxa that differed across locations in the office, see Data Set S8 in the supplemental material.

10.1128/mSystems.00022-16.8 Data Set S8  Differentially abundant OTUs across office locations, as determined by ANCOM. Download Data Set S8, XLSX file, 0.05 MB. Copyright © 2016 Chase et al. 2016 Chase et al. This content is distributed under the terms of the Creative Commons Attribution 4.0 International license.

Interestingly, within a location, the pattern of differences among samples varied between floors and walls/ceilings. With unweighted UniFrac, floor samples were more similar to other floor samples than wall/ceiling samples were to other wall/ceiling samples (Fig. 2). In contrast, with weighted UniFrac, floor samples were less similar to one another. Thus, community differences among floor samples were driven primarily by differences in the relative abundance of the same operational taxonomic units (OTUs), while the differences among wall/ceiling samples were driven more by the presence or absence of particular OTUs. The same pattern was statistically significant in all of the cities but strongest in Flagstaff (see Fig. S6 in the supplemental material).

### Sampling disrupts microbial communities detectably, but the effect is small.

Each sampling plate in this study contained at least two rows of sampling swatches (Fig. 1b). During each of the four sampling periods, the “frequently sampled” row of materials was sampled every other day, while the “infrequently sampled” row was sampled every 3 weeks. Through this design, we could detect differences in bacterial community richness and composition between frequently and infrequently sampled materials (Fig. 1c and 3; see Fig. S3 in the supplemental material). Comparing pairs of frequently and infrequently sampled sites showed that the samples from infrequently sampled surfaces were richer, as measured by PD, than the frequently sampled surfaces (Monte Carlo *t* test, 3.75; *P* = 0.0002; *n* = 412) (Fig. 1 and 3A). This sampling frequency effect was the strongest in floor samples (Fig. 3A). While differences in bacterial community composition between frequently and infrequently sampled rows existed, they were not statistically significant (weighted UniFrac, PERMANOVA, *P* = 0.109, pseudo-*F* = 1.63; unweighted UniFrac, PERMANOVA, *P* = 0.175, pseudo-*F* = 1.13) (Fig. 3B and C). For the specific taxa that were most different between frequently and infrequently samples sites, see Data Set S9 in the supplemental material.

10.1128/mSystems.00022-16.3 Figure S3  Disturbance due to repeat sampling, though detectable, is small compared to that due to other variables. This is an analog of Fig. 3 illustrating fungal community results. Download Figure S3, TIF file, 12.8 MB. Copyright © 2016 Chase et al. 2016 Chase et al. This content is distributed under the terms of the Creative Commons Attribution 4.0 International license.

10.1128/mSystems.00022-16.9 Data Set S9  Differentially abundant OTUs across frequently and infrequently sampled sites, as determined by ANCOM. Download Data Set S9, XLSX file, 0.01 MB. Copyright © 2016 Chase et al. 2016 Chase et al. This content is distributed under the terms of the Creative Commons Attribution 4.0 International license.

We note that although this sampling frequency effect is present, it is small compared to the effects of biological interest in our study. Figure 3B and C clearly show that the compositional differences that we observed as a result of sampling frequency are much smaller than those based on other effects, such as the season or the location where the sampling plate was installed (i.e., the floor, ceiling, or wall), which are statistically significant.

### Cities and offices have signature microbial communities, though the effect is less pronounced in offices.

As in previous studies of office bacterial communities (8), we observed that *Proteobacteria*, *Firmicutes*, and *Actinobacteria* were the three dominant phyla across all of the locations in all of the offices and in all of the cities. To investigate the extent to which offices have city-specific bacterial communities (i.e., offices within a given city have bacterial communities that look more like the communities in other offices in the same city than those in offices in other cities), we developed both support vector machine (SVM) and random forests machine learning classifiers in an attempt to classify the city from which a microbiome sample was derived, as described in reference 18. Though the two approaches gave similar results, the SVM classifiers performed slightly better on the basis of F-1 scores (a weighted average of precision and recall ranging between 0 and 1).

**FIG 4:**
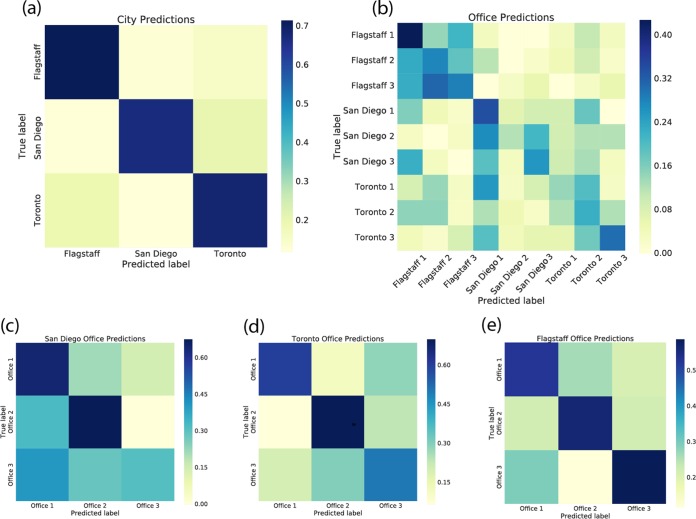
Confusion matrices illustrating the performance of city and office SVM classifiers. (a) True (actual) city and predicted city for our SVM classifier when trained and tested on office microbiomes by city. Dark colors on the diagonal indicate that the predicted label is very frequently correct. (b) True and predicted offices for our SVM classifier when trained and tested on office microbiomes from all of the cities labeled by individual offices. Note that the diagonal is not as dark as in panel a, illustrating that the predicted office labels are not correct as often as the predicted city labels. When an incorrect office is predicted, it is often in the correct city, as indicated by the darker colors surrounding the diagonal. (c to e) True and predicted offices for our SVM classifiers when trained and tested on office microbiomes from cities individually.

With SVM, we were able to predict the city of origin of unlabeled samples (where we knew the city of origin but withheld that information from the classifier) with 85% accuracy only on the basis of its microbiome composition (Fig. 4; F-1 score, 0.85; see Fig. S5 in the supplemental material). The SVM model correctly classified the city and performed 2.67 times as well as random guessing (where the most common city was always chosen).

10.1128/mSystems.00022-16.5 Figure S5  Confusion matrices illustrating the performance of city and office SVM classifiers. This is an analog of Fig. 4 illustrating fungal community results. Download Figure S5, TIF file, 4.8 MB. Copyright © 2016 Chase et al. 2016 Chase et al. This content is distributed under the terms of the Creative Commons Attribution 4.0 International license.

**FIG 5:**
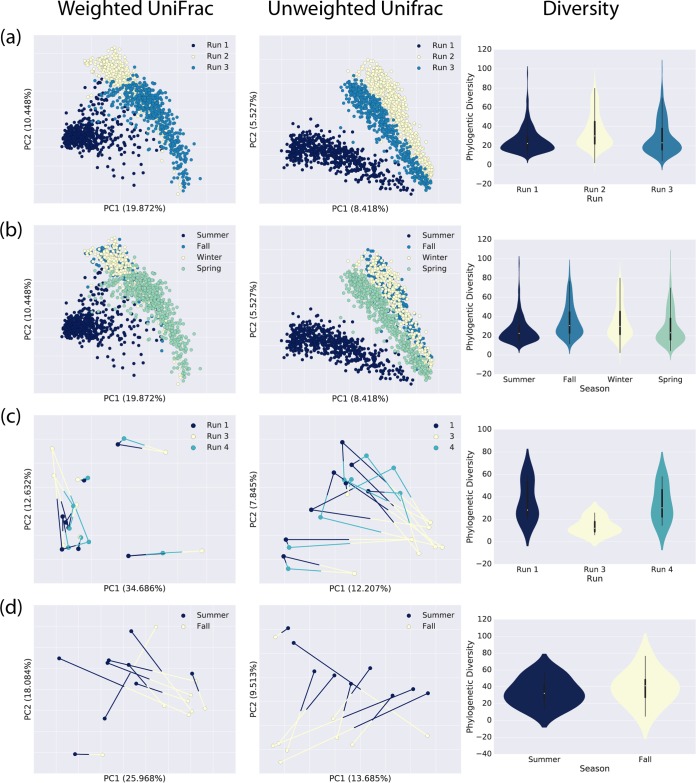
Investigation of the effect of the sequencing run on the observed bacterial community composition. (a) Weighted and unweighted UniFrac PCoA and PD by sequencing run for sequencing runs 1 to 3. (b) Weighted and unweighted UniFrac PCoA and PD by season. (c) Weighted and unweighted UniFrac PCoA and PD for samples at the same sites in summer and fall based on a single sequencing run. (d) Weighted and unweighted UniFrac PCoA and PD of technical replicate samples sequenced in sequencing runs 1, 3, and 4.

10.1128/mSystems.00022-16.6 Figure S6  Investigation of the effect the sequencing run on the observed bacterial community composition. This is an analog of Fig. 5 illustrating fungal community results. Download Figure S6, TIF file, 3 MB. Copyright © 2016 Chase et al. 2016 Chase et al. This content is distributed under the terms of the Creative Commons Attribution 4.0 International license.

A potential confounding effect related to the city-specific microbial communities was the effect of office-specific bacterial communities. Because each city contained three offices, it is possible that our classifier actually predicts individual offices, because each office is found in only one city. If the bacteria found in each office were primarily derived from the inhabitants of the office, who are known to have highly personalized microbiomes (19, 20), we would expect to observe office-specific effects (8). To determine if individual offices were the source of the city-specific bacterial communities that we observed, we trained classifiers to predict the office from which a sample was taken. For all nine offices, our best classifiers achieved an F-1 score of 0.28 and a mean accuracy 2.022 times that achieved by random guessing. Evaluation of the confusion matrix (Fig. 4b) shows that the majority of misclassifications happen when the classifier assigns an incorrect office in the correct city, further suggesting that offices within cities look more similar to each other than to offices in different cities. Our design of including multiple offices in multiple cities therefore allows us to separate city-specific bacterial community effects from office-specific bacterial community effects, and our findings suggest that communities differ across cities but not necessarily across the offices in those cities.

If we train the same classifiers only on offices in individual cities, the classifiers can predict the office of origin more accurately but still less accurately than they can predict the city of origin (Flagstaff offices only, *F* − 1 = 0.41; San Diego offices only, *F* − 1 = 0.37; Toronto offices only, *F* − 1 = 0.53; Fig. 4c to e). This is especially interesting because even within a city, offices were different from each other, for example, in terms of size, usage patterns, and ventilation systems, suggesting that geography is more important than any of these features in driving the bacterial community composition of the offices.

In addition to the community composition differences that are shown by SVM, Flagstaff offices had richer communities (as determined by PD) than San Diego or Toronto (two-sample Monte Carlo *t* test with 999 permutations, test statistic = 7.029, *P* = 3.411e-12 and test statistic = 9.156, *P* = 0.0, respectively), which were more similar to one another in community richness (two-sample Monte Carlo *t* test with 999 permutations, test statistic = 2.352, *P* = 0.019) (Fig. 1). For the specific taxa that were most different across cities, see Data Set S10 in the supplemental material. Together, these differences enable the classifiers to differentiate cities.

10.1128/mSystems.00022-16.10 Data Set S10  Differentially abundant OTUs across cities, as determined by ANCOM. Download Data Set S10, XLSX file, 0.1 MB. Copyright © 2016 Chase et al. 2016 Chase et al. This content is distributed under the terms of the Creative Commons Attribution 4.0 International license.

### Office bacterial communities are moderately influenced by indirect contact with humans.

In addition to our office surface samples, we collected human skin, nasal, oral, and fecal microbiome samples from 11 inhabitants of one of our Flagstaff offices and from the individuals performing the sampling in all three cities. Using SourceTracker2 (Biota Technology, Houston, TX) (21), we defined the human microbiome samples as potential “sources” and the office surface samples as “sinks.” This allowed us to determine which human subjects, and which body sites of those subjects, might serve as sources for the microbes found on the office surfaces.

Across all nine offices, human skin bacterial communities were the largest identifiable source of the office bacterial community samples, with at least 25 to 30% of the office surface microbiome being derived from human skin (see Fig. 7A). The human nasal microbiome also appeared to be a small but consistent source of office surface microbial communities. The largest source of microbial communities in these offices was nonhuman (the unknown proportion; see Fig. 7A).

We next defined the source samples as skin from the individuals who collected the samples in each city and 11 inhabitants of Flagstaff office 1 (some of whom worked in that office for only part of the year). Our goal was to determine whether the personalized skin microbiomes (20) of the individuals working in an office were drivers of the office bacterial communities or whether the office bacterial communities looked more generically like human skin (see Fig. 7B). The surface bacterial communities from office 1 in Flagstaff do not appear to be more derived from the inhabitants of that office than do those of the other offices in this study. Similarly, the office bacterial communities do not appear to be influenced more by the individual who sampled in that city (who wore gloves during sampling) than do those in other cities by the individuals who sampled there or those in the Flagstaff 1 office by its inhabitants. Thus, it appears that the personalized microbiomes of the office inhabitants or samplers were not transferred to our office surfaces.

Other work has shown that the personalized human microbiome is transferred from human subjects to their offices through direct contact (13, 14). In our study, we specifically requested that office inhabitants not touch the materials and we required our samplers to wear gloves while collecting samples. We therefore suspect that our inability to detect a personalized human microbiome signal in our office microbiome data is a result of our sampling swatches not being in direct contact with the office inhabitants, though the surfaces still look more generically like human skin, suggesting a role for indirect transfer of skin microbes to office surfaces.

### The sequencing run can be an important confounding factor in long-term temporal studies of bacterial communities.

Our bacterial samples were sequenced in three sequencing runs to facilitate the sequencing of the large number of samples collected in this study and to provide us with a way to begin analyzing data before all of the samples were collected. We were specifically interested in using the samples collected early in this study to inform decisions that would be made during later sampling periods, such as how frequently we should collect samples to track changes in the office bacterial communities. As the cost of sequencing continues to decline, we expect that this model will become increasingly frequent. An issue with this approach, however, is that it conflates time with the sequencing run, potentially introducing a batch effect. Samples collected during the summer sampling period were sequenced in our first sequencing run, samples collected during the fall and winter periods were sequenced in our second run, and samples collected during the spring period were sequenced in our third sequencing run (Fig. 5a and b).

To detect and quantify the run effect, the same eight samples (our technical replicates) were sequenced in three runs: the run of summer samples (run 1), the run of spring samples (run 3), and an additional partial run used to understand interrun variation (run 4). (Run 2 contained our fall and winter samples but did not contain these eight technical replicate samples.) Theoretically, these eight samples should have been identical in both composition and richness across the three runs. While we expected differences between sequencing runs, the observed variation between runs was larger than we expected. The technical replicates sequenced in run 3 (spring samples), in particular, were very different in composition (with unweighted UniFrac) and richness (PD) from those sequenced in runs 1 and 4, but all of these runs were significantly different from one another on the basis of these metrics (Fig. 5D). With weighted UniFrac, none of the runs were significantly different from the others. The larger differences observed with unweighted UniFrac than with weighted UniFrac suggest that the run effect we observe is due primarily to differences in low-abundance taxa and not to shifts in the dominant taxa between runs.

We attempted several approaches for removing this run effect. First, we ensured that the sequence length was the same across all of the runs by trimming longer reads (251 bases) to the same length as our shortest reads (151 bases). All of the data presented here are based on these length-normalized reads. We additionally tried various filtering strategies, including filtering out low-abundance OTUs and filtering out the OTUs that were the most differentially abundant across the technical replicates (as identified by analysis of the composition of microbiomes [ANCOM] [22]), where *n* (the number of OTUs filtered out) was varied between 0 and 1,000. While the variation across runs was reduced by these strategies, we did not observe significant differences in the results until we filtered out >1,000 of the differentially abundant OTUs, and even at this level, the run effect was only minimally reduced.

Despite the differences in replicates across runs, there is evidence of seasonal variation in the bacterial communities. Figure 5C illustrates differences between the bacterial community compositions of two samples each from the same 10 sampling swatches, in the summer and fall. These samples were sequenced in the same run, so there is no confounding run effect. In weighted and unweighted UniFrac principal-coordinate analysis (PCoA), the samples collected during the same season cluster with one another, suggesting that these are more similar to each other than are samples collected from the same swatches but in different seasons. Similarly, the fall samples had higher community richness than the summer samples when sequenced in the same run (Fig. 5). Taken together, Fig. 5c and d suggest that the observed seasonal differences are likely representative of the underlying biology though conflated with a run effect. We note that we were able to determine this only because our experimental design involved repeated sequencing of samples. This is essential for studies that require sequencing to be split across multiple runs.

### Community richness of office surfaces is consistent over time and impacted by sampling.

Our summer sampling period, which took place immediately after UV sterilization of the plates, showed that bacterial communities were less rich than the fall or winter sampling periods that followed (see Fig. 8). The spring sampling period subsequently appears to be less rich, on average, than the summer, fall, or winter sampling period. While these data suggest that community richness increases following the installation of plates, plateaus, and begins to decrease, we note that the summer, fall/winter, and spring samples were sequenced on sequencing runs 1, 2, and 3, respectively, and that these sequencing runs differed in their mean richness (Fig. 5d), so as described above, this is likely a combination of real biological effects and run effects.

For an additional illustration of the divergence of the richness of the floor samples and the wall/ceiling samples, with the distributions becoming increasingly more bimodal with time, see Fig. 8. The upper mode in these distributions is composed primarily of floor samples, whereas the lower mode is composed of wall/ceiling samples.

### Community compositions were not found to be associated with any indoor or material environmental covariates.

**FIG 6:**
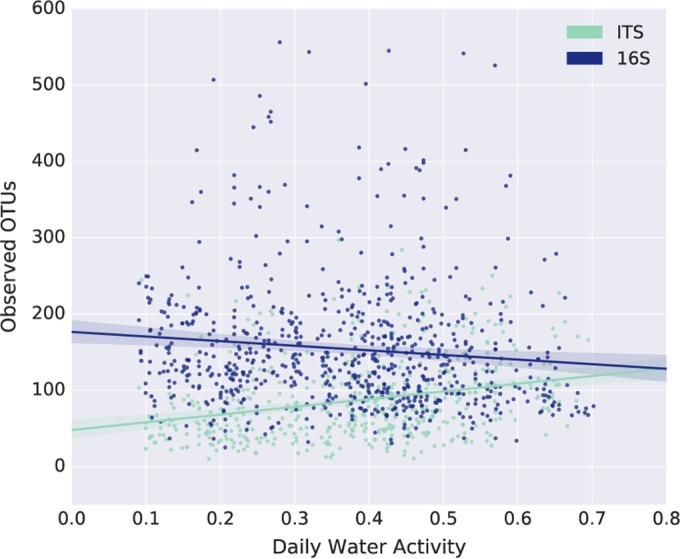
Community richness as a function of equilibrium relative humidity of sites for bacterial (16S) and fungal (ITS) communities. We found weak correlations between the community richness and equilibrium relative humidity (ERH) of the sites. While these correlations are statistically significant, we do not find these relationships to be convincing, for reasons discussed in Results (also see Text S4 in the supplemental material).

Throughout this study, indoor environment as well as surface microenvironment (i.e., sensors specifically collecting data from our sampling swatches) data were collected, including surface and air temperatures, equilibrium relative humidity, relative humidity, room occupancy, and visible-light illumination. Despite the extent of these environmental data and a considerable effort to identify associations between microbial composition and these data, we failed to identify significant associations between building science and microbiome data. For example, there were no meaningful correlations between levels of community richness, composition, or abundance of specific taxa of interest and equilibrium relative humidity of the material (the humidity at which moisture is no longer being absorbed by or evaporated from the material). The single exception to this was a weak but significant positive correlation between fungal community richness and equilibrium relative humidity (*r* = 0.315, *P* = 3.24e-12) (Fig. 6). A significant correlation between bacterial community richness and equilibrium relative humidity was also observed, but this was weaker and negative, so we suspect that it is a false-positive result. For details of additional tests we performed to detect associations between the office microbiome and environmental parameters (though none of these tests yielded significant associations), see Text S4 in the supplemental material.

## DISCUSSION

Our experimental design allowed us to isolate variables that were confounded in previous studies such as surface material, human usage patterns, and location in an office (15, 20, 23). It is important to note that the goals of those studies did not necessarily require separation of these variables, so our findings are not in disagreement with theirs. Our ability to better explore these parameters in isolation has led to a better understanding of the microbiology of the BE and enables us to make recommendations that can improve future studies.

Our data suggest that the personalized office microbiomes that have previously been reported are more likely to be the result of direct human contact with the surfaces sampled, rather than the result of climatic or other differences in the offices themselves.

**FIG 7:**
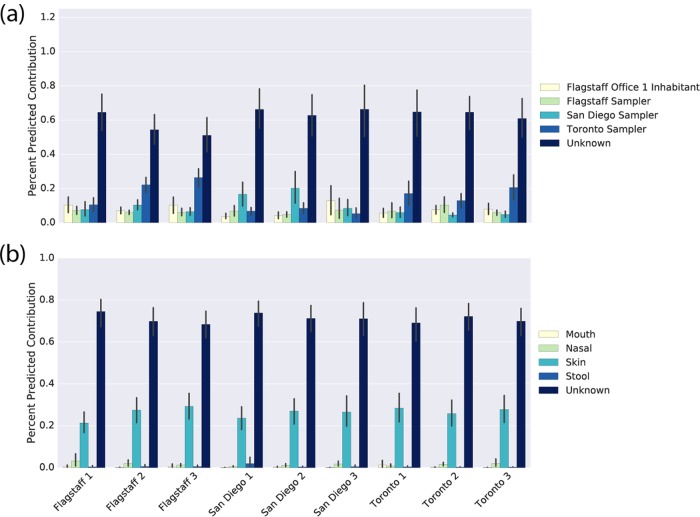
Human-based source tracking of office microbiome samples. (a) Percent contributions of the microbiomes of different sites of the human body to the office bacterial communities. Unknown indicates contribution from a source other than the body sites tested here. (b) Percent contribution of microbiomes of different human subjects to the office bacterial communities. The unknown contribution is from a source other than the individuals tested here.

Many of the studies that report that office microbial communities are personalized (i.e., that individual offices have signature microbial communities) have sampled materials that were already present in the offices in areas of high human traffic (9, 10, 13) rather than materials installed for the specific purpose of microbial tracking. Our sampling materials were specifically placed in low-traffic areas and included signs requesting that individuals not touch the materials. While we did observe an office-specific microbial community effect, the size of the effect was smaller than what has been previously reported and smaller than the city-specific bacterial community effect that we observed (Fig. 4b). This suggests that “personalized office microbiomes” are likely largely a result of the “personalized human microbiomes” (19) of the office inhabitants, based on the microbes that they leave behind on surfaces through direct interaction. The source of the city-specific BE microbial communities that we observe will be important to understand better, as it could, for example, result in city-specific “cage effects” in murine microbiome studies. We observed that at least 25 to 30% of the office surface microbiomes were human derived (Fig. 7), primarily from human skin, suggesting that indirect contact does impact office microbiomes (e.g., through the office inhabitants’ “personal microbial clouds” [24]), but this is far less than what would be expected on surfaces with which the inhabitants are in direct contact (14, 20).

Our nested experimental design has allowed us to draw several conclusions that should impact the design of future BE microbiome studies. First, the sampling of different locations (such as the floor and ceiling in multiple locations) in a BE is likely to result in the detection of greater variability among microbial communities than the sampling of different surface materials (e.g., carpet versus tile flooring). Thus, limited sampling effort is likely better expended on the sampling of different locations rather than of different materials. Next, it is likely more useful to sample offices in buildings in different climates than to sample multiple offices in the same city, as there seem to be consistent differences in the compositions of the offices we studied by city. Finally, sampling of bacterial communities with dry swabs (like those used here) is sufficient to obtain consistent bacterial community 16S rRNA profiles by Illumina sequencing. Sampling of these communities does “disturb” them, but the effect size of those disturbances will likely be small relative to the biological effects of interest. Our approach of dry swabbing did not work as well for sampling of fungal communities (we received very low PCR yields; see Text S4 in the supplemental material), though it has been successfully applied in previous work that used the same swabs (25). We expect that this differential success is a result of differences in biomass (public rest room floor tiles were previously sampled by swabbing a larger area with much heavier human traffic). Additional work should be performed to understand how collection of fungal samples can be performed on low-biomass BE samples.

**FIG 8:**
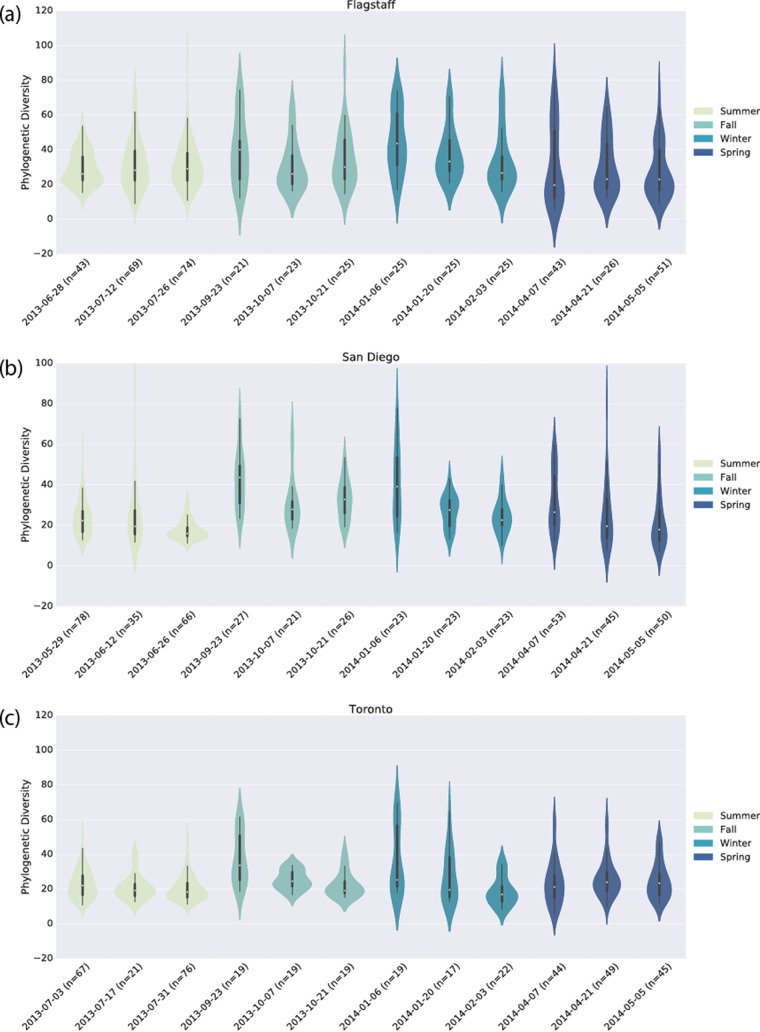
Longitudinal analysis of bacterial PD over 1 year in Flagstaff (a), San Diego (b), and Toronto (c). Each “violin” represents a 2-week period at the beginning, in the middle, or at the end of our four sampling periods.

Despite considerable effort, we did not detect any difference in microbial communities associated with material or indoor environment covariates such as temperature or equilibrium relative humidity. While this does not prove that these parameters do not impact microbial communities, it does suggests that the variation across indoor environmental conditions, which are restricted to a narrow range for the comfort of the inhabitants, may not be enough to drive changes in the microbial communities. Another compatible and viable hypothesis is that rather than observing a succession of microbial communities over the course of the year, we instead observed the passive accumulation of biologically inactive microbes. This is consistent with the relatively small amount of change in these communities over the year, as illustrated in Fig. 8. BE surface microbial communities may behave similarly to those found in the soils of the Atacama Desert (26), waiting for liquid water to become active. Our findings suggest that detailed monitoring of material and indoor environment covariates is not necessary in studies of the composition of the microbiology of BEs, except perhaps when the parameters are likely to be microbially relevant (such as the addition of liquid water through real or simulated flooding) or fall far outside the normal range. This information may, however, be important for understanding bacterial and/or fungal loads on BE surfaces (25, 27).

Our experimental design included incremental sampling over a period of 1 year. Because of the large number of samples that were collected and to allow us to analyze data as they accumulated to make decisions about future sampling, it was not possible to include all of our samples in a single Illumina MiSeq run. As shown in Fig. 5, this resulted in a sequencing run artifact in our data. As microbiome sequencing becomes more routine, for example, as we transition toward human microbiome monitoring in clinical settings to discover early signals of dysbioses, this type of design is likely to become more common and sequencing run effects will need to be understood, controlled for, and (ideally) eradicated. We note that in this study we were working with low-biomass samples and relatively small biological effect sizes, so the possibility of a run effect interfering with biological effects of interest was likely amplified. However, our approach to detecting and understanding this run effect was useful here, and we recommend that this be done routinely. Specifically, we suggest that in studies necessitating multiple sequencing runs, researchers include either some of their samples (as we did here) or control samples (such as artificial communities of known composition) in all sequencing runs as technical replicates. An accumulation of publicly available sequence data replicated across sequencing runs will help us to understand, and possibly develop methods to control for, sequencing run effects. As a starting point, these data could be used to understand whether approaches that have been developed for controlling batch effects in gene expression and microarray experiments (28, 29) would be useful in sequence-based microbiome studies.

This work expands our basic understanding of the factors impacting the microbiology of BEs. The human skin microbiome has a considerable impact on the composition of microbiomes on BE surfaces, even when humans are not in direct contact with those surfaces. Further, within the range of human comfort, differences in the indoor environment do not appear to impact microbial community composition. These findings provide insight into what drives the composition of BE microbiomes, and taking the results together, we suspect that in the absence of extreme conditions (for example, flooding), microbes may be passively accumulating on surfaces rather than undergoing an active succession process. We additionally show that features previously suspected to be important in driving the microbial composition of BE surfaces, such as the surface material, do not seem to impact community composition under typical circumstances. As we continue to expand our understanding of the microbiology of the BE, possibly including routine monitoring of microbial communities as indicators of changes toward communities that may impact human health, the results presented here will help with making critical decisions about important dependent and independent variables in future research efforts.

## MATERIALS AND METHODS

### City and office selection.

To develop our understanding of how microbes establish themselves in BEs over time and a range of building parameters, we sampled nine offices in three cities over the period of 1 year. We selected three cities that were climatically different from one another, as determined by their Köppen climate classifications, i.e., San Diego, CA, which has an arid Mediterranean climate (Köppen classification, Bsh or Csa/Csb); Flagstaff, AZ, which has a semiarid climate (Köppen classification, Dsb/Csb); and Toronto, ON, which has a humid continental climate (Köppen classification, Dfa/Dfb).

In each of these three cities, we selected three offices with the goal of making these offices as similar as possible to one another. Offices were always shared spaces, meaning that they had more occupancy than just a single individual but had controlled access. All of the offices had similar patterns of usage, in that they tended to have the highest occupancy on weekdays during business hours. We selected offices that had consistent ventilation rates and were approximately the same size. Finally, we excluded older buildings that contained laboratory space. For a summary of the parameters of these offices, see Table S7 in the supplemental material. Human subject research was performed in accordance with University of Colorado Institutional Review Board protocol 12-0582 and University of California San Diego Human Research Protection Program protocol 141853.

### Sampling plate construction and installation.

In each office, we installed three sampling plates, one on the floor, one on the ceiling, and one on the wall, as illustrated in Fig. 1a (which is a photograph showing the three installed sampling plates in a single office). The plates were constructed from sheets of birch plywood measuring 600 by 600 by 6 mm. Swatches of each sampling material (painted drywall, ceiling tile, and carpet tile) were mounted to the back of the plate and exposed through holes in the plates. Each plate contained a minimum of two swatches of each material that were sampled for microbial community composition. One of each of these swatches was sampled frequently (as often as every 2 days during our sampling periods), and the other was sampled infrequently (every 3 weeks during our sampling periods) to evaluate the impact of sampling on the microbial communities. Our wall-mounted plates (i.e., those not on the floor or ceiling) contained an additional three swatches of each material that were monitored for equilibrium relative humidity on the surfaces. These swatches were not sampled for microbial composition, as the equilibrium relative humidity sensor covered most of the available space. The relative location of each material was consistent across all of the plates.

### Environmental monitoring.

Each plate contained an Onset HOBO UX90-005 occupancy sensor and an Onset HOBO U12-012 temperature, relative humidity, and light sensor. The occupancy sensors monitored occupancy by sensing movement up to approximately 5 m away from the plate. The wall plates additionally contained a Decagon EM-50 data logger with three VP3 sensors that measured equilibrium relative humidity and near-surface temperatures (one on each material). In one office in each city, an additional two VP3 sensors were used to measure the same conditions on the drywall samples on the floor and ceiling. All measurements were recorded every 5 min, except for the occupancy sensors, which recorded the time of each change of state (e.g., going from occupied to unoccupied). Data were downloaded from the data loggers once a month with the HOBOware 3.5.0 and the Decagon ECH_2_O Utility software packages.

### Plate sterilization.

The plates were constructed in Toronto and then shipped to San Diego State University to be sterilized in order to maintain consistent sterilization techniques. We hoped to get all of the surfaces as close as possible to the same starting point to provide a baseline to compare subsequent samples. Any DNA present in the first poststerilization sampling event could be controlled for as DNA present prior to the experiment start. UV sterilization was selected because of its ability to denature DNA by cross-linking nucleotides and creating thymine dimers while not damaging the surface material. The sampling portion of the plate containing the six or nine office materials was covered with Saran Wrap and sealed around the edges with packing tape. The back of the plate was also sealed with Saran Wrap. Both sides of the plate were sterilized under UV light for 10 min. This sterilization procedure was repeated for all 27 plates. Plates were swabbed before sealing and again (at time zero) when the Saran Wrap was first removed at installation time in each of the nine offices, to determine whether UV sterilization worked as expected.

### Swabbing procedure.

The plates were shipped sealed in Saran Wrap as described above. Immediately after the plates were unwrapped, each surface was swabbed. This was done to ensure that the surfaces were sterile or, if they were not sterile, to establish the starting community on each surface. Once the plates were installed, the regular sampling schedule was initiated. Sampling was done with BD CultureSwab sterile swabs. Each swab tube was labeled with a unique sample ID and corresponding bar code. The swab was removed from the tube immediately prior to swabbing. The cotton swab was swiped left to right across the surface, moving downward (or toward the individual who was performing the swabbing), for approximately 3 s. The swab was turned 180°, and the process was repeated starting from the bottom and moving upward. Once the swabbing was complete, the swab was returned to the sterile tube and the next swab was removed to swab the next surface. Once all of the surfaces were swabbed, the tubes were placed in a −20°C freezer for storage.

### Sampling.

Sampling was performed in four 6-week periods, one period per season (summer, fall, winter, and spring). The first day of sampling in San Diego was 29 May 2013; in Flagstaff, it was 27 June 2013; and in Toronto, it was 3 July 2013. Samples were taken every other day in the first sampling period and every Monday, Wednesday, and Friday in the subsequent periods to simplify collection. The last three sampling periods took place simultaneously in all of the cities (this was not possible for the first sampling period, as we had one team member travel to each city to ensure consistent experimental setup, and our first sampling period had to begin immediately after removal of the Sara Wrap). Period 2 was 9/23/2013 to 11/4/2013, period 3 was 1/6/2014 to 2/17/2014, and period 4 was 4/7/2014 to 5/19/2014.

### Sequencing and data analysis.

Samples were collected during four 6-week sampling periods, one per season. The samples were labeled on the basis of the cual-id labeling protocol (30). 16S rRNA gene sequencing was used to profile bacterial communities. Human bacterial microbiome samples were collected and processed through collaboration with American Gut (31).

The V4 hypervariable region of the 16S rRNA gene was amplified by barcoded PCR with primers 515F (GTGCCAGCMGCCGCGGTAA) and 806R (GGACTACHVGGGTWTCTAAT) in accordance with the Earth Microbiome Project protocol (32). All sequencing was performed at Argonne National Laboratories on an Illumina MiSeq. Raw FASTQ files containing sequence data for both the 3′ and 5′ reads and the bar codes were provided by the sequencing facility via the secure file transfer protocol. The bacterial raw sequence data contained 41,335,672 DNA reads for the three runs. The sequence length for the first two sequencing runs was 251 bases, and it was 151 bases for the third sequencing run. All reads were trimmed to exactly 151 bases before analysis so they could be directly compared.

All bioinformatic analysis was performed with the 5′ reads. The reads were demultiplexed and assigned to sample IDs with QIIME 1.9.1 (33), and quality filtering was performed as described in reference 34. After demultiplexing and quality filtering, 33,799,179 reads remained.

The sequences were assigned to OTUs with QIIME’s uclust-based (35) open-reference OTU picking protocol (36) and the Greengenes 13_8 reference sequence set (37) at 97% similarity. The centroid of each OTU was chosen as the representative sequence for the OTU. OTU representative sequences were aligned with PyNAST (38), and phylogenetic trees were constructed with FastTree (39) for PD calculations. Taxonomy was assigned to sequences with QIIME’s uclust consensus taxonomy assigner (40). The resulting bacterial OTU table contained 2,309 samples, with a median of 7,849 sequences per sample.

Beta diversity calculations were performed with QIIME’s implementations of weighted and unweighted UniFrac (16) by using exactly 1,000 randomly selected sequences per sample (41). Samples with <1,000 sequences were not included in the calculations. Community richness was calculated by using PD (17) and compared across categories with a nonparametric *t* test with 999 permutations. Comparisons of significantly different OTUs across sample groups were performed with scikit-bio (scikit-bio.org) by ANCOM (22). For all permutation-based tests, the nested structure of the experimental design was respected so that after the label shuffling, all further nested observations continued to be grouped together.

The SVM analysis to predict the city on the basis of sample OTU features was performed with a linear kernel by randomly splitting the data into two halves (training and test sets), tuning the SVM with 5-fold cross-validation on the training set, and then observing the prediction error rates on the test set. SVM and random forests machine analyses were run by using the implementations of these methods from scikit-learn (42).

Differentially abundant OTUs across plate locations, frequently/infrequently rows, and cities were identified by ANCOM (22) (see Data Sets S8 to S10 in the supplemental material, respectively). All analyses were run with jupyter Notebooks (jupyter.org). The notebooks used in this project are all available at the GitHub website (https://github.com/gregcaporaso/office-microbes). Raw sequence data are publicly accessible in the European Read Archive under accession number ERP014651.

## References

[1] EPA 1989 Report to Congress on indoor air quality, volume II: assessment and control of indoor air quality. EPA/400/1-89/001C. Environmental Protection Agency, Washington, DC.

[2] Klepeis NE, Nelson WC, Ott WR, Robinson JP, Tsang AM, Switzer P, Behar JV, Hern SC, Engelmann WH 2001 The National Human Activity Pattern Survey (NHAPS): a resource for assessing exposure to environmental pollutants. J Expo Anal Environ Epidemiol 11:231–252. doi:10.1038/sj.jea.7500165. 11477521

[3] Husman T 1996 Health effects of indoor-air microorganisms. Scand J Work Environ Health 22:5–13. doi:10.5271/sjweh.103. 8685674

[4] Lee L, Tin S, Kelley ST 2007 Culture-independent analysis of bacterial diversity in a child-care facility. BMC Microbiol 7:27. doi:10.1186/1471-2180-7-27. 17411442 PMC1853100

[5] Kembel SW, Jones E, Kline J, Northcutt D, Stenson J, Womack AM, Bohannan BJ, Brown GZ, Green JL 2012 Architectural design influences the diversity and structure of the built environment microbiome. ISME J 6:1469–1479. doi:10.1038/ismej.2011.211. 22278670 PMC3400407

[6] Ortega-Calvo JJ, Hernandez-Marine M, Sáiz-Jiménez C 1991 Biodeterioration of building materials by cyanobacteria and algae. Int Biodeterior 28:165–185. doi:10.1016/0265-3036(91)90041-O.

[7] Viitanen H, Vinha J, Salminen K, Ojanen T, Peuhkuri R, Paajanen L, Lähdesmäki K 2010 Moisture and bio-deterioration risk of building materials and structures. J Build Phys 33:201–224. doi:10.1177/1744259109343511.

[8] Hewitt KM, Gerba CP, Maxwell SL, Kelley ST 2012 Office space bacterial abundance and diversity in three metropolitan areas. PLoS One 7:e37849. doi:10.1371/journal.pone.0037849. 22666400 PMC3364274

[9] Hewitt KM, Mannino FL, Gonzalez A, Chase JH, Caporaso JG, Knight R, Kelley ST 2013 Bacterial diversity in two neonatal intensive care units (NICUs). PLoS One 8:e54703. doi:10.1371/journal.pone.0054703. 23372757 PMC3553055

[10] Flores GE, Bates ST, Knights D, Lauber CL, Stombaugh J, Knight R, Fierer N 2011 Microbial biogeography of public restroom surfaces. PLoS One 6:e28132. doi:10.1371/journal.pone.0028132. 22132229 PMC3223236

[11] Mole B 23 5 2013 Patients leave a microbial mark on hospitals: the microscopic ecosystem in health-care centres mirror those of their patients. Nat News.

[12] Shogan BD, Smith DP, Packman AI, Kelley ST, Landon EM, Bhangar S, Vora GJ, Jones RM, Keegan K, Stephens B, Ramos T, Kirkup BC Jr., Levin H, Rosenthal M, Foxman B, Chang EB, Siegel J, Cobey S, An G, Alverdy JC, Olsiewski PJ, Martin MO, Marrs R, Hernandez M, Christley S, Morowitz M, Weber S, Gilbert J 2013 The Hospital Microbiome Project: meeting report for the 2nd Hospital Microbiome Project, Chicago, USA, January 15(th), 2013. Stand Genomic Sci 8:571–579. doi:10.4056/sigs.4187859. 24501640 PMC3910697

[13] Lax S, Smith DP, Hampton-Marcell J, Owens SM, Handley KM, Scott NM, Gibbons SM, Larsen P, Shogan BD, Weiss S, Metcalf JL, Ursell LK, Vázquez-Baeza Y, Van Treuren W, Hasan NA, Gibson MK, Colwell R, Dantas G, Knight R, Gilbert JA 2014 Longitudinal analysis of microbial interaction between humans and the indoor environment. Science 345:1048–1052. doi:10.1126/science.1254529. 25170151 PMC4337996

[14] Lax S, Hampton-Marcell JT, Gibbons SM, Colares GB, Smith D, Eisen JA, Gilbert JA 2015 Forensic analysis of the microbiome of phones and shoes. Microbiome 3:21. doi:10.1186/s40168-015-0082-9. 25969737 PMC4427962

[15] Meadow JF, Altrichter AE, Kembel SW, Moriyama M, O’Connor TK, Womack AM, Brown GZ, Green JL, Bohannan BJ 2014 Bacterial communities on classroom surfaces vary with human contact. Microbiome 2:7. doi:10.1186/2049-2618-2-7. 24602274 PMC3945812

[16] Lozupone CA, Hamady M, Kelley ST, Knight R 2007 Quantitative and qualitative beta diversity measures lead to different insights into factors that structure microbial communities. Appl Environ Microbiol 73:1576–1585. doi:10.1128/AEM.01996-06. 17220268 PMC1828774

[17] Faith DP 1992 Conservation evaluation and phylogenetic diversity. Biol Conserv 61:1–10. doi:10.1016/0006-3207(92)91201-3.

[18] Knights D, Costello EK, Knight R 2011 Supervised classification of human microbiota. FEMS Microbiol Rev 35:343–359. doi:10.1111/j.1574-6976.2010.00251.x. 21039646

[19] Califf K, Gonzalez A, Knight R, Caporaso JG 2014 The human microbiome: getting personal. Microbe 9:410–415. http://caporasolab.us/assets/pdf/califf-et-al-final.pdf.

[20] Fierer N, Lauber CL, Zhou N, McDonald D, Costello EK, Knight R 2010 Forensic identification using skin bacterial communities. Proc Natl Acad Sci U S A 107:6477–6481. doi:10.1073/pnas.1000162107. 20231444 PMC2852011

[21] Knights D, Kuczynski J, Charlson ES, Zaneveld J, Mozer MC, Collman RG, Bushman FD, Knight R, Kelley ST 2011 Bayesian community-wide culture-independent microbial source tracking. Nat Methods 8:761–763. doi:10.1038/nmeth.1650. 21765408 PMC3791591

[22] Mandal S, Van Treuren W, White RA, Eggesbø M, Knight R, Peddada SD 2015 Analysis of composition of microbiomes: a novel method for studying microbial composition. Microb Ecol Health Dis 26:27663. 26028277 10.3402/mehd.v26.27663 PMC4450248

[23] Dunn RR, Fierer N, Henley JB, Leff JW, Menninger HL 2013 Home life: factors structuring the bacterial diversity found within and between homes. PLoS One 8:e64133. doi:10.1371/journal.pone.0064133. 23717552 PMC3661444

[24] Meadow JF, Altrichter AE, Bateman AC, Stenson J, Brown GZ, Green JL, Bohannan BJ 2015 Humans differ in their personal microbial cloud. PeerJ 3:e1258. doi:10.7717/peerj.1258. 26417541 PMC4582947

[25] Fouquier J, Schwartz T, Kelley ST 30 12 2015 Rapid assemblage of diverse environmental fungal communities on public restroom floors. Indoor Air doi:10.1111/ina.12279. 26717555

[26] Neilson JW, Quade J, Ortiz M, Nelson WM, Legatzki A, Tian F, LaComb M, Betancourt JL, Wing RA, Soderlund CA, Maier RM 2012 Life at the hyperarid margin: novel bacterial diversity in arid soils of the Atacama Desert, Chile. Extremophiles 16:553–566. doi:10.1007/s00792-012-0454-z. 22527047

[27] Liu CM, Aziz M, Kachur S, Hsueh P-R, Huang Y-T, Keim P, Price LB 2012 BactQuant: an enhanced broad-coverage bacterial quantitative real-time PCR assay. BMC Microbiol 12:56. doi:10.1186/1471-2180-12-56. 22510143 PMC3464140

[28] Johnson WE, Li C, Rabinovic A 2007 Adjusting batch effects in microarray expression data using empirical Bayes methods. Biostatistics 8:118–127. doi:10.1093/biostatistics/kxj037. 16632515

[29] Lauss M, Visne I, Kriegner A, Ringnér M, Jönsson G, Höglund M 2013 Monitoring of technical variation in quantitative high-throughput datasets. Cancer Inform 12:193–201. doi:10.4137/CIN.S12862. 24092958 PMC3785384

[30] Chase JH, Bolyen E, Rideout JR, Caporaso JG 2016 cual-id: globally unique, correctable, and human-friendly sample identifiers for comparative omics studies. mSystems 1:e00010-15. doi:10.1128/mSystems.00010-15. PMC5069752 27822516

[31] American Gut Project. http://americangut.org/.

[32] Caporaso JG, Lauber CL, Walters WA, Berg-Lyons D, Huntley J, Fierer N, Owens SM, Betley J, Fraser L, Bauer M, Gormley N, Gilbert JA, Smith G, Knight R 2012 Ultra-high-throughput microbial community analysis on the Illumina HiSeq and MiSeq platforms. ISME J 6:1621–1624. doi:10.1038/ismej.2012.8. 22402401 PMC3400413

[33] Caporaso JG, Kuczynski J, Stombaugh J, Bittinger K, Bushman FD, Costello EK, Fierer N, Peña AG, Goodrich JK, Gordon JI, Huttley GA, Kelley ST, Knights D, Koenig JE, Ley RE, Lozupone CA, McDonald D, Muegge BD, Pirrung M, Reeder J, Sevinsky JR, Turnbaugh PJ, Walters WA, Widmann J, Yatsunenko T, Zaneveld J, Knight R 2010 QIIME allows analysis of high-throughput community sequencing data. Nat Methods 7:335–336. doi:10.1038/nmeth.f.303. 20383131 PMC3156573

[34] Bokulich NA, Subramanian S, Faith JJ, Gevers D, Gordon JI, Knight R, Mills DA, Caporaso JG 2013 Quality-filtering vastly improves diversity estimates from Illumina amplicon sequencing. Nat Methods 10:57–59. doi:10.1038/nmeth.2276. 23202435 PMC3531572

[35] Edgar RC 2010 Search and clustering orders of magnitude faster than BLAST. Bioinformatics 26:2460–2461. doi:10.1093/bioinformatics/btq461. 20709691

[36] Rideout JR, He Y, Navas-Molina JA, Walters WA, Ursell LK, Gibbons SM, Chase J, McDonald D, Gonzalez A, Robbins-Pianka A, Clemente JC, Gilbert JA, Huse SM, Zhou H-W, Knight R, Caporaso JG 2014 Subsampled open-reference clustering creates consistent, comprehensive OTU definitions and scales to billions of sequences. PeerJ 2:e545. doi:10.7717/peerj.545. 25177538 PMC4145071

[37] McDonald D, Price MN, Goodrich J, Nawrocki EP, DeSantis TZ, Probst A, Andersen GL, Knight R, Hugenholtz P 2012 An improved Greengenes taxonomy with explicit ranks for ecological and evolutionary analyses of bacteria and archaea. ISME J 6:610–618. doi:10.1038/ismej.2011.139. 22134646 PMC3280142

[38] Caporaso JG, Bittinger K, Bushman FD, DeSantis TZ, Andersen GL, Knight R 2010 PyNAST: a flexible tool for aligning sequences to a template alignment. Bioinformatics 26:266–267. doi:10.1093/bioinformatics/btp636. 19914921 PMC2804299

[39] Price MN, Dehal PS, Arkin AP 2010 FastTree 2—approximately maximum-likelihood trees for large alignments. PLoS One 5:e9490. doi:10.1371/journal.pone.0009490. 20224823 PMC2835736

[40] Bokulich NA, Rideout JR, Kopylova E, Bolyen E, Patnode J, Ellett Z, McDonald D, Wolfe B, Maurice CF, Dutton RJ, Turnbaugh PJ, Knight R, Caporaso JG 2015 A standardized, extensible framework for optimizing classification improves marker-gene taxonomic assignments. PeerJ PrePrints 3:e1502. doi:10.7287/peerj.preprints.934v2.

[41] Lozupone C, Knight R 2005 UniFrac: a new phylogenetic method for comparing microbial communities. Appl Environ Microbiol 71:8228–8235. doi:10.1128/AEM.71.12.8228-8235.2005. 16332807 PMC1317376

[42] Pedregosa F, Varoquaux G, Gramfort A, Michel V, Thirion B, Grisel O, Blondel M, Prettenhofer P, Weiss R, Dubourg V, Vanderplas J, Passos A, Cournapeau D, Brucher M, Perrot M, Duchesnay É 2011 Scikit-learn: machine learning in python. J Mach Learn Res 12:2825–2830.

