# Supplementary figures and images for: Geography and Location Are the Primary Drivers of Office Microbiome Composition
Source: mSystems. 2016 Apr 19;1(2):e00022-16. doi: 10.1128/mSystems.00022-16 (PMC5069741; doi:10.1128/mSystems.00022-16)

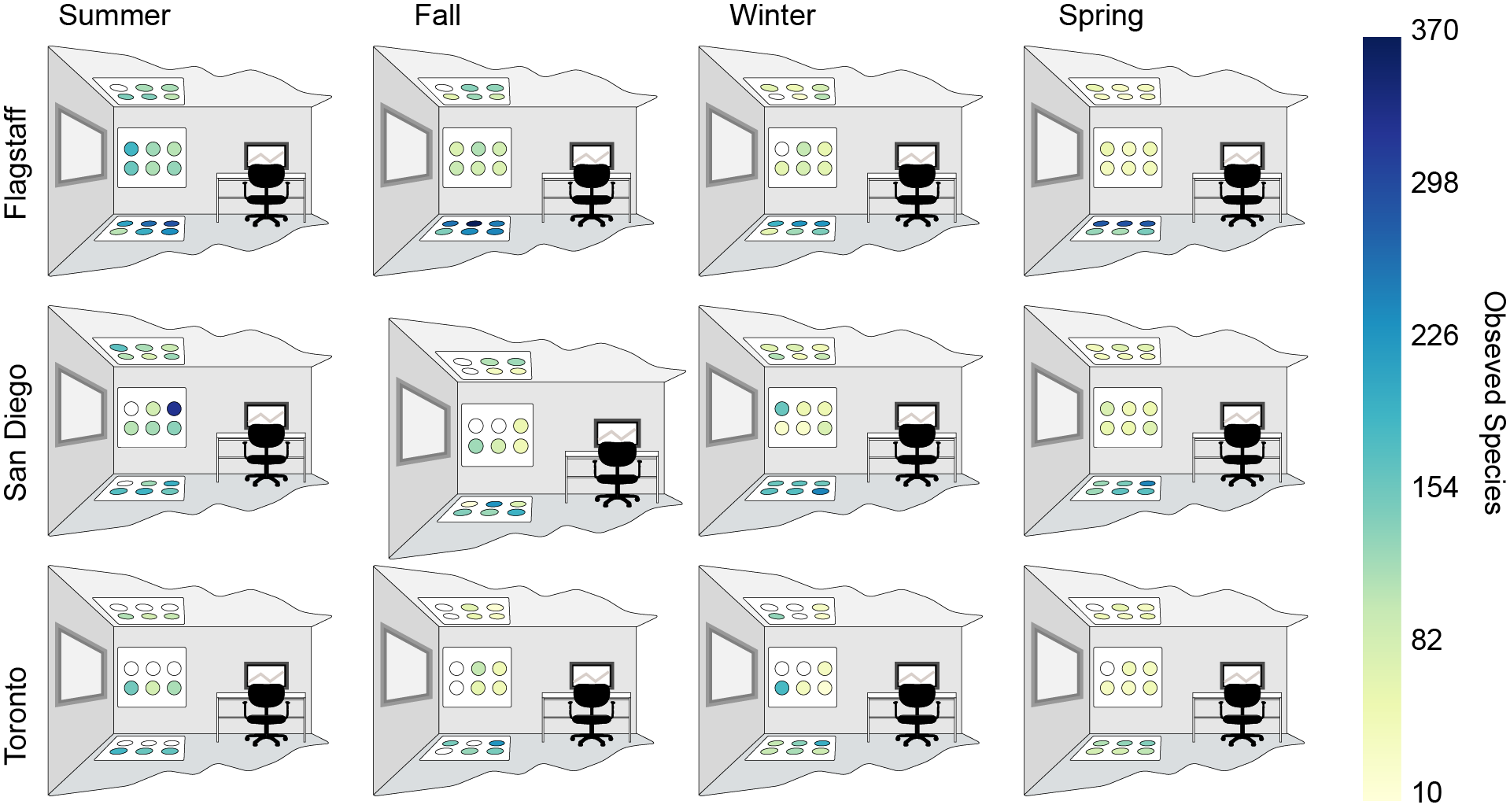

Supplement: Figure S1 [file sys002162014sf1.tif]

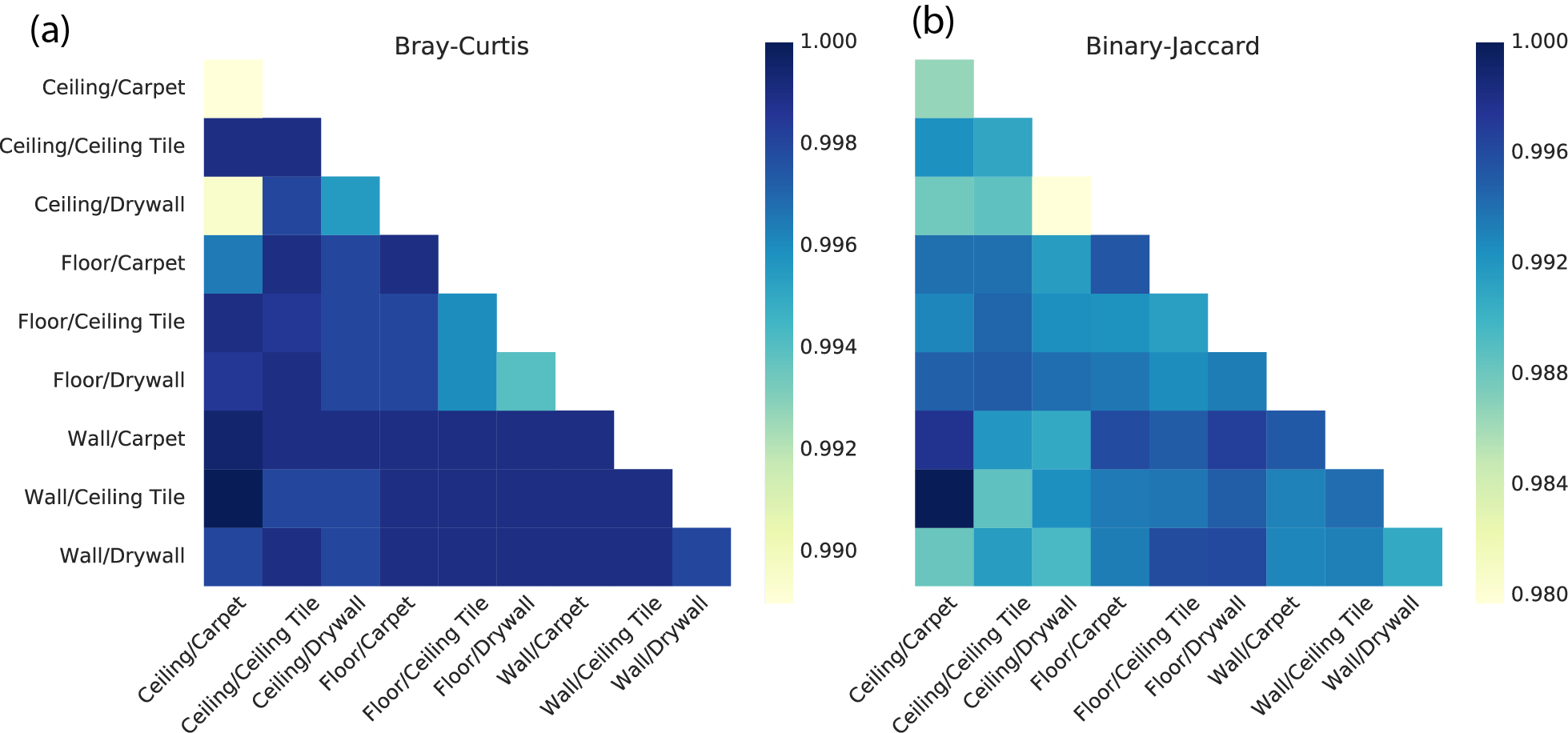

Supplement: Figure S2 [file sys002162014sf2.tif]

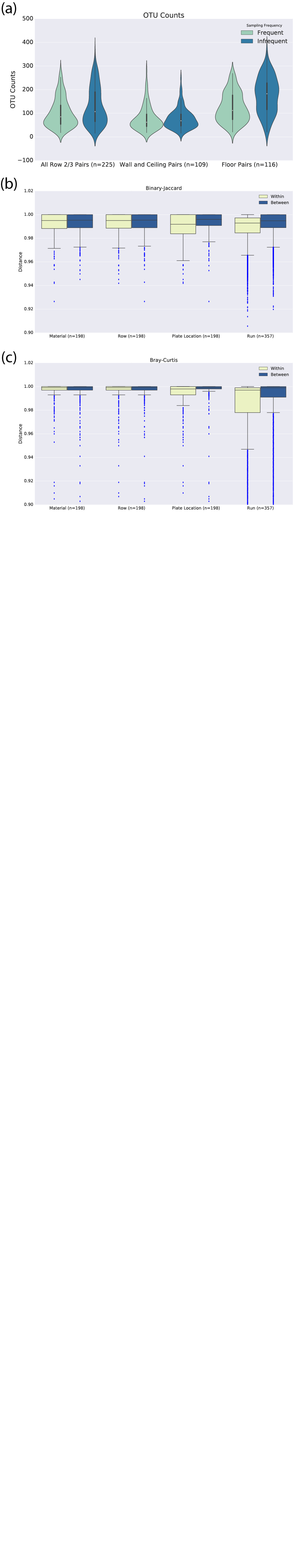

Supplement: Figure S3 [file sys002162014sf3.tif]

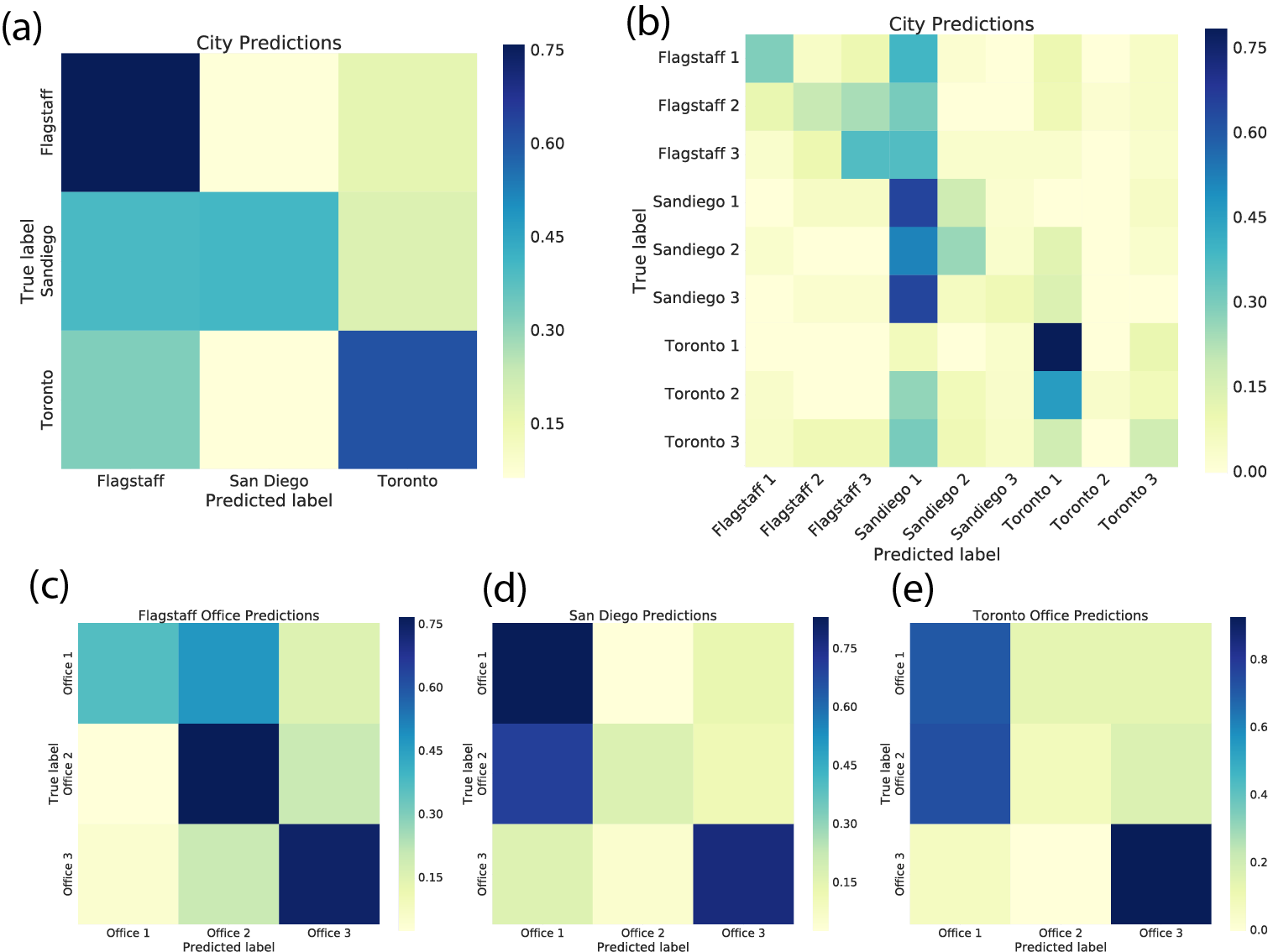

Supplement: Figure S5 [file sys002162014sf5.tif]

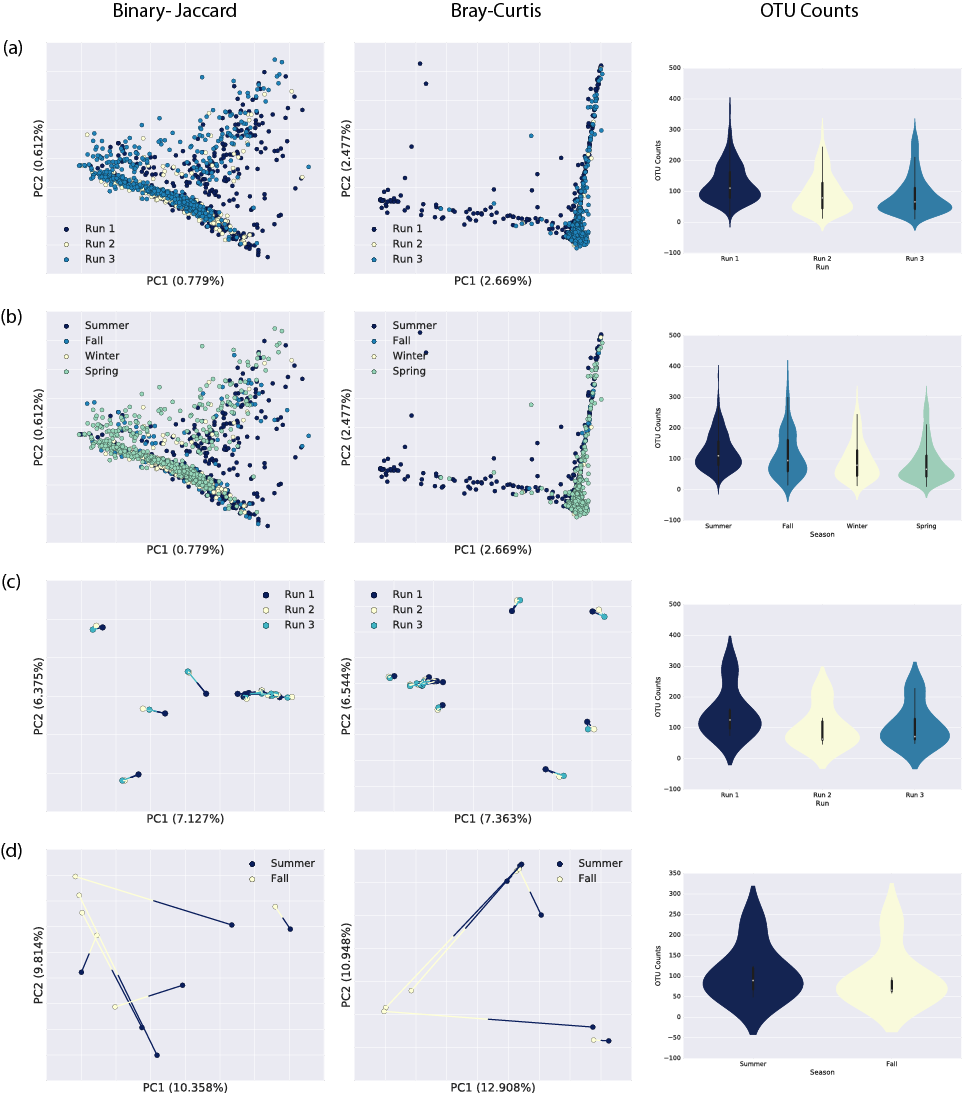

Supplement: Figure S6 [file sys002162014sf6.tif]
