# Supplementary material for: Geography and Location Are the Primary Drivers of Office Microbiome Composition
Source: mSystems. 2016 Apr 19;1(2):e00022-16. doi: 10.1128/mSystems.00022-16 (PMC5069741; doi:10.1128/mSystems.00022-16)
Supplement: Text S4 [file sys002162014s4.docx]

# Supplementary Results

## **Community compositions were not found to be associated with any indoor or material environmental covariates**

In addition to the analyses described in *Results*, we employed many other tests to identify associations between the office microbiome composition and indoor and material environment covariates. We tested for correlation between microbiome characteristics and building science data based on single timepoints of building science data (e.g. the equilibrium relative humidity measurement at the time of sampling) but also extrapolated this to equilibrium relative humidity measurements from the hour immediately prior to when a sample was taken, as well as data from the week and the month prior. We followed a similar approach with temperature and illumination, though even with multiple different types of comparisons we did not observe any significant associations. To further investigate possible relationships, we looked specifically between offices which exhibited large differences in building measurements. One of the largest differences in building data was observed in San Diego during the summer period. During this period, one San Diego office saw a “time of wetness” (the amount of time a given surface had a equilibrium relative humidity measurement over a certain percent) over 60% of ~2500 hours while a second San Diego office saw a time of wetness less than 10 hours. If a single building measurement were associated with microbial community differences, we expected that this would be a likely candidate. Despite the large differences in equilibrium relative humidity, similar differences in community composition and richness did not exist (two-sample monte carlo t-test with 999 permutations, test-statistic = -1.419, p-value = 0.158). Additionally, of the taxa that were identified to be the most different across cities, the one most significantly correlated with equilibrium relative humidity, the order *Sphingomonadales,* did not show significant differences between these two San Diego offices, suggesting that its correlation with equilibrium relative humidity may be due to chance. Further analysis including interactions between building parameters, building parameters and sample metadata, log functions, and higher order interactions evaluated with a Lasso regression also did not reveal any discernable patterns. There are several possible explanations for these lack of associations, but one likely important factor is that all of the offices in the study were quite consistent in important factors: they were generally occupied based on the traditional work week, they were all mechanically heated and cooled to similar temperature and relative humidity conditions, they had similar occupant densities, and none experienced extreme conditions, such as a moisture problem, over the course of the investigation.

## **Fungal results**

In addition to the 16S rRNA, we sequenced the ITS-1 region of the fungal genome (a non-functional region between the 18S and 5.8S rRNA genes). Many of the patterns that we report for bacterial community composition and richness were also observed for fungal communities, but there was large variability in the quality and quantity of fungal sequence data. We suspect that this may be due to an inability to collect sufficient fungal biomass from surfaces using the dry swabbing technique we employed in this study, or issues with DNA extraction from fungi as has been recently observed.

Our fungal community taxonomic profiles are similar to those previously reported in the BE. For example, the most dominant taxa identified by [(1)](https://paperpile.com/c/ArRedQ/VQar), Ascomycota (phylum) and Basidiomycota (phylum), were also the highest abundance identified phyla in our study, representing 32.8% and 17.1% of our fungal community composition. Our most dominant classes, Eurotiomycetes, Dothideomycetes, and Agaricomycetes, respectively, were present in high abundance by [(1)](https://paperpile.com/c/ArRedQ/VQar). They report that 59% of their OTUs could not be taxonomically assigned at the phylum level, while 45% (a similar proportion) of our fungal OTUs could be assigned taxonomy at the phylum level.

The mean and median Bray-Curtis distance between all fungal samples following open reference OTU were 0.991 and 0.999, respectively, suggesting little to no overlap in the composition of the communities on these surfaces. These distances are much higher than what has been previously reported [(2, 3)](https://paperpile.com/c/ArRedQ/2etK6+7oZN5). Closed reference OTU picking results in lower mean (0.949) and median (0.998) distances, however these are still significantly higher distances than what has been observed in other studies. We present our observations from these data here, but are cautious to avoid over-interpretation of what may be spurious associations.

As in bacterial communities, fungal communities had lower richness in all offices in the summer sampling period than in all subsequent periods (two-sample monte carlo t-test with 999 permutations, p-values less than 0.05 for all comparisons, except for Flagstaff summer and fall, and Toronto summer and fall). This is likely due to the summer sampling period starting immediately after the sampling materials were sterilized. Floor samples had significantly higher richness in all cities, in all periods (two-sample monte carlo t-test with 999 permutations, p-values less than 0.05 for all comparisons, except for San Diego fall and, and Toronto spring; Supplementary Figure 1). When comparing the Flagstaff fall and winter samples (which were sequenced in the same sequencing run), the floor samples were significantly different in composition than the wall/ceiling samples (Permanova with Bray-Curtis pseudo-F=1.099, p-value = 0.015; Binary-Jaccard pseudo-F = 1.11431, p-value < 0.001) however sample composition did not differ with surface material (Permanova with Bray-Curtis pseudo-F = 1.01254, p-value = 0.308; Binary-Jaccard pseudo-F = 1.01402, p-value = 0.199).

Sampling frequency resulted in noticeable but not statistically significant differences in fungal community composition (Supplementary Figure 3) for paired samples from frequently and infrequently sampled rows (Permanova with Bray-Curtis pseudo-F=1.06308, p-value = 0.092; Binary-Jaccard pseudo-F=1.0128, p-value = 0.279). As with bacterial communities, this effect was small relative to the biologically relevant effects observed (Supplementary Figure 3).

We were able to distinguish cities using SVMs (Supplementary Figure 4), however the accuracy of the predictions was lower than those for the bacterial communities ([F-1 score 0.65](https://docs.google.com/presentation/d/1B_slt59vK7DFQ6BCy51LuEjCD1zgwj6oQknXXD5jdE0/edit#slide=id.gf2cec6224_0_51)). The SVM classifier performed as well in predicting office ([F-1 score 0.](https://docs.google.com/presentation/d/1B_slt59vK7DFQ6BCy51LuEjCD1zgwj6oQknXXD5jdE0/edit#slide=id.gf2cec6224_0_51)29) with the fungal data as with bacterial data. In the context of previous research [(4)](https://paperpile.com/c/ArRedQ/J3Jfh) this suggests that higher quality ITS sequence data could provide a powerful vehicle for predicting geographic origin of samples.

While we did observe a run effect in fungal communities, this effect was not as strong as for the bacterial data (Figure 5, Supplementary Figure 5). As with the other effects presented here, due to the large average distance between samples, it would be not be appropriate to extrapolate this finding to future studies.

# Supplementary Methods

## Fungal sequencing

ITS-1 (the non-coding region between the 18S and 5.8S rRNA genes) sequencing was used to profile fungal communities using the ITS1-F (CTTGGTCATTTAGAGGAAGTAA) and ITS2 (GCTGCGTTCTTCATCGATGC) primers. The fungal raw sequence data contained 39,173,141 reads for the three runs. After demultiplexing and quality filtering, 30,516,432 fungal reads remained.

All bioinformatics analysis was performed on the 5’ reads as described in *Methods*, unless otherwise noted here. The sequences were assigned to operational taxonomic units (OTUs) using QIIME’s uclust-based [(5)](https://paperpile.com/c/ArRedQ/nIjPN), open-reference OTU picking protocol [(6)](https://paperpile.com/c/ArRedQ/fpXmv) with the UNITE [(7)](https://paperpile.com/c/ArRedQ/Fi3f4) ver6_dynamic_s_30.12.2014 reference set at 97% similarity. ghost-tree was tested for phylogenetic diversity calculations [(8)](https://paperpile.com/c/ArRedQ/CsJ6x), however ghost-tree currently supports closed-reference OTUs only. Due to the low number of our fungal reads that hit the reference database, we chose to focus our ITS analyses on open-reference OTUs and non-phylogenetic diversity metrics. The fungal OTU table contained 2151 samples with a median of 1987 sequences per sample.

Beta diversity calculations were performed using QIIME’s implementations Bray-Curtis [(9)](https://paperpile.com/c/ArRedQ/1BNEi) and Jaccard distance with exactly 1000 randomly selected sequences per sample [(10)](https://paperpile.com/c/ArRedQ/prQyi). Samples with less than 1000 sequences were not included in the calculations. Community richness was computed using Observed OTUs.

# References

1. [**Fouquier J**, **Schwartz T**, **Kelley ST**. 2015. Rapid assemblage of diverse environmental fungal communities on public restroom floors. Indoor Air.](http://paperpile.com/b/ArRedQ/VQar)

2. [**Bell TH**, **El-Din Hassan S**, **Lauron-Moreau A**, **Al-Otaibi F**, **Hijri M**, **Yergeau E**, **St-Arnaud M**. 2014. Linkage between bacterial and fungal rhizosphere communities in hydrocarbon-contaminated soils is related to plant phylogeny. ISME J **8**:331–343.](http://paperpile.com/b/ArRedQ/2etK6)

3. [**Willger SD**, **Grim SL**, **Dolben EL**, **Shipunova A**, **Hampton TH**, **Morrison HG**, **Filkins LM**, **O’Toole GA**, **Moulton LA**, **Ashare A**, **Sogin ML**, **Hogan DA**. 2014. Characterization and quantification of the fungal microbiome in serial samples from individuals with cystic fibrosis. Microbiome **2**:40.](http://paperpile.com/b/ArRedQ/7oZN5)

4. [**Grantham NS**, **Reich BJ**, **Pacifici K**, **Laber EB**, **Menninger HL**, **Henley JB**, **Barberán A**, **Leff JW**, **Fierer N**, **Dunn RR**. 2015. Fungi identify the geographic origin of dust samples. PLoS One **10**:e0122605.](http://paperpile.com/b/ArRedQ/J3Jfh)

5. [**Edgar RC**. 2010. Search and clustering orders of magnitude faster than BLAST. Bioinformatics **26**:2460–2461.](http://paperpile.com/b/ArRedQ/nIjPN)

6. [**Rideout JR**, **He Y**, **Navas-Molina JA**, **Walters WA**, **Ursell LK**, **Gibbons SM**, **Chase J**, **McDonald D**, **Gonzalez A**, **Robbins-Pianka A**, **Clemente JC**, **Gilbert JA**, **Huse SM**, **Zhou H-W**, **Knight R**, **Caporaso JG**. 2014. Subsampled open-reference clustering creates consistent, comprehensive OTU definitions and scales to billions of sequences. PeerJ **2**:e545.](http://paperpile.com/b/ArRedQ/fpXmv)

7. [**Kõljalg U**, **Nilsson RH**, **Abarenkov K**, **Tedersoo L**, **Taylor AFS**, **Bahram M**, **Bates ST**, **Bruns TD**, **Bengtsson-Palme J**, **Callaghan TM**, **Others**. 2013. Towards a unified paradigm for sequence-based identification of fungi. Mol Ecol **22**:5271–5277.](http://paperpile.com/b/ArRedQ/Fi3f4)

8. [**Fouquier J**, **Rideout JR**, **Bolyen E**, **Chase J**, **Shiffer A**, **McDonald D**, **Knight R**, **Caporaso JG**, **Kelley ST**. 2016. ghost-tree: creating hybrid-gene phylogenetic trees for diversity analyses. Microbiome **4**:1–10.](http://paperpile.com/b/ArRedQ/CsJ6x)

9. [**Beals EW**. 1984. Bray-Curtis ordination: an effective strategy for analysis of multivariate ecological data. Adv Ecol Res **14**:1–55.](http://paperpile.com/b/ArRedQ/1BNEi)

10. [**Lozupone C**, **Knight R**. 2005. UniFrac: a new phylogenetic method for comparing microbial communities. Appl Environ Microbiol **71**:8228–8235.](http://paperpile.com/b/ArRedQ/prQyi)
